# A Matter of the Heart: The African Clawed Frog *Xenopus* as a Model for Studying Vertebrate Cardiogenesis and Congenital Heart Defects

**DOI:** 10.3390/jcdd3020021

**Published:** 2016-06-04

**Authors:** Annemarie Hempel, Michael Kühl

**Affiliations:** Institute of Biochemistry and Molecular Biology, Ulm University, Albert-Einstein-Allee 11, 89081 Ulm, Germany; annemarie.hempel@uni-ulm.de

**Keywords:** *Xenopus*, heart development, congenital heart defects, morpholino, model organism

## Abstract

The African clawed frog, *Xenopus,* is a valuable non-mammalian model organism to investigate vertebrate heart development and to explore the underlying molecular mechanisms of human congenital heart defects (CHDs). In this review, we outline the similarities between *Xenopus* and mammalian cardiogenesis, and provide an overview of well-studied cardiac genes in *Xenopus*, which have been associated with congenital heart conditions. Additionally, we highlight advantages of modeling candidate genes derived from genome wide association studies (GWAS) in *Xenopus* and discuss commonly used techniques.

## 1. Advantages of *Xenopus* as a Model Organism

In the last decades, studies in amphibians such as the African clawed frog *Xenopus laevis* have substantially contributed to deciphering the mechanisms of early vertebrate heart development. *Xenopus* and humans share various anatomical, physiological as well as genetic similarities [1,2] suggesting that they have comparable underlying gene regulatory networks [1,3]. Several features make *Xenopus* an attractive model for studying cardiovascular development and disease. First, *Xenopus* husbandry is simple and breeding can be done year-round by inducing females to spawn after injection of human chorionic gonadotropin. The litter size can be as high as 2000 eggs per day per frog and embryos can be obtained by *in vitro* fertilization [3]. In addition, *in vitro* fertilization allows the synchronization of embryonic development between sibling embryos. Developmental stages of *Xenopus laevis* embryogenesis have been described and well characterized [4]. Second, the eggs are rather large, about 1.2 mm in diameter, which makes them suitable for surgical micromanipulation. Third, the extrauterine development of the embryos and detailed fate-maps [5,6,7] allow tissue-specific manipulations like microinjections or mechanistic analysis in explant assays. Fourth, the partial transparency of the embryos makes them amenable to imaging of the heart. Lastly, early embryos are capable of developing to early tadpole stages in the absence of a working circulation system. This is advantageous because, unlike in mammalian models, it enables the study of early cardiovascular defects *in vivo* that are not complicated by secondary effects resulting from lack of circulation.

The aforementioned advantages of using *Xenopus* as a model system are complemented by various molecular techniques. Detailed gene expression maps of molecular cardiac markers for the specific cardiac lineages and the different stages of cardiogenesis are available [8] and enable researchers to monitor gene expression in functional studies by *in situ* hybridization. Additionally, immunohistochemistry assays can be applied to analyze gene products at the protein level. Regarding functional assays, antisense morpholino oligonucleotides (MOs), cardiac explants and transgenesis are currently the main tools in *Xenopus* research (see next section). With respect to MO based strategies and novel genome editing approaches, it is noteworthy to mention that *Xenopus laevis* is tetraploid whereas the related *Xenopus tropicalis* is diploid.

## 2. Tool Kit for Studying Heart Development and Diseases in *Xenopus*

*Xenopus* embryos are particularly amenable to microinjections and microsurgery allowing functional assessment of a gene of interest. Genes can be over-expressed by injection of *in vitro* synthesized mRNA [9], while morpholino oligonucleotides (MOs) approaches offer the possibility to easily generate loss-of-function phenotypes. MOs are highly stable, non-toxic, synthetic nucleic acid analogues, which are not degraded by endogenous nucleases [10,11,12]. MOs hybridize to their complementary mRNA, thereby blocking translation or interfering with mRNA splicing. MOs have several benefits (reviewed in [13,14,15]) that include tissue-specific loss-of-function studies with dose-response effects. MOs can target multiple gene products allowing examination of functional gene redundancy. Additionally, multiple knockdowns by combinational MO injections combined with corresponding rescue experiments can unravel epistatic relationships of interacting genes. Since MOs target maternal and zygotic mRNAs simultaneously, they can be used to analyze maternal gene products as long as no corresponding maternal protein has been deposited. Furthermore, the Stainier group reported that morphants do not appear to induce gene compensatory mechanisms to the same extent as mutants do [16]. Recently, the specificity of MOs has been controversially discussed, especially in the zebrafish field [17,18]. However, several guidelines for control experiments [13,19] can be adhered to, which should ensure MO specificity and may prevent some of the MOs’ pitfalls. Thus, MO applications were and still are a valuable tool for *Xenopus* research and have enabled vast progress in understanding the molecular mechanisms during development in the last two decades.

A fundamental technique to study molecules with respect to their function in heart development is the use of explanted tissue from *Xenopus* embryos [14,15,20]. These explants include dorsal marginal zone (DMZ), ventral marginal zone (VMZ) and animal cap explants. During normal embryogenesis, heart precursor cells are specified bilaterally adjacent to the Spemann organizer in the mesodermal germ layer on the dorsal side. Therefore, explants of this region can be used to test for factors that impede cardiac tissue development and to gain deeper insights into the underlying molecular mechanisms directing cardiogenesis [21,22,23,24]. In contrast, the ventral mesoderm in *Xenopus* develops into blood. Accordingly, VMZ explants can be used to test the ability of factors to induce cardiogenesis from non-cardiac specified tissue [21,23,25]. Finally, explants of the animal cap represent a population of pluripotent progenitor cells [14]. Thus, animal caps can be used to analyze which transcription factors and signaling molecules influence cardiac gene expression and differentiation [20].

In addition to MO and tissue explant techniques, the use of transgenic frogs allows for investigating promoters and tissue-specific gene expression. The first approaches to generating transgenic frogs involved microinjections of DNA resulting in mosaic animals [26]. A few years later, techniques such as Restriction Enzyme Mediated Integration, REMI, through sperm nuclei transplantation or the use of transposable elements, like the *Tol2* transposon, were used to establish transgenic frogs (reviewed in [14,15,27]).

The advent of new genome editing tools such as the clustered regulatory inter-spaced short palindromic repeat (CRISPR)/Cas system extend the available toolbox for *Xenopus* [28,29]. First data derived from *X. tropicalis* show that the majority of CRISPR/Cas9 derived mutants phenocopy MO knockdowns [28]. Unpublished data from *Xenopus laevis* also suggest promising advances as well, however, the future will show how amenable this technique is.

Finally, cardiac anatomical morphologies can be easily visualized by histological sections and standard microscopy techniques or by *in vivo* imaging. Advancements in imaging technology such as in echocardiography [30] potentially allow not only 3D but also 4D *in vivo* imaging of the *Xenopus* heart in the future, thus enabling more detailed analyses of the morphological changes during heart development and onset of cardiac defects.

## 3. *Xenopus* as a Model for Vertebrate Cardiogenesis

The development of the vertebrate heart is a highly conserved, well-orchestrated process that involves cell specification and differentiation along with extensive morphogenetic remodeling of the cardiac tissue. Early cardiogenesis has been analyzed in *Xenopus* through a combination of fate mapping approaches [5,6,7], transplant experiments [24,31], gene expression analyses [8], histological sections and whole mount confocal microscopy or immunohistochemistry and 3D reconstruction [32,33]. While mammals and birds have a four-chambered heart consisting of two atria and two ventricles, amphibians have a three-chambered heart with a single ventricle, which resembles the mammalian left ventricle [8]. Thus, the *Xenopus* heart represents the evolutionary intermediate between the two-chambered fish heart and the four-chambered hearts of birds and mammals. In this section, we summarize the most important steps of *Xenopus* and murine heart development and highlight the similarities and differences that can be taken advantage of when studying vertebrate cardiogenesis.

**Figure 1:**
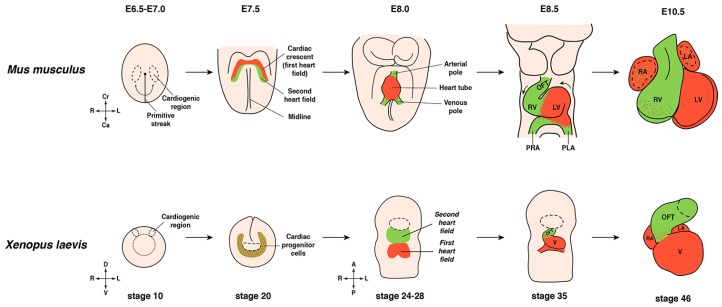
Early cardiogenesis in mouse (upper panel) and *Xenopus* (lower panel). During gastrulation myocardial progenitor cells arise from the mesoderm and migrate to the ventral midline to the anterior part of the embryo. The progenitor cells form the cardiac crescent (first heart field), which already includes differentiated cardiomyocytes. The primary heart tube is formed at the ventral midline, starts to beat and undergoes looping and further morphological remodeling. Subsequently, the different cardiac chambers are formed. Cr-Ca/D-V/A-P and R-L axes are indicated. A: anterior; Ca, caudal; Cr: cranial; D: dorsal; L: left; LA: left atrium; LV: left ventricle; OFT: outflow tract; PLA: primitive left atrium; P: posterior; PRA: primitive right atrium; R: right; RA: right atrium; RV: right ventricle; V: ventral.

Vertebrate cardiogenesis (Figure 1, Table 1) begins at the onset of gastrulation (*Xenopus* stage 10, mouse E6.5). The heart originates from the precardiac mesoderm that is located bilaterally on the dorsal side of the *Xenopus* embryo on either side of the Spemann’s organizer. In mouse embryos, the cardiac primordia lie on opposite sides of the cranial midline [31,34,35,36]. Gastrulation movements cause the cardiac progenitors to migrate anteriorly to the ventral midline.

The common cardiac progenitor cells fuse at the ventral midline immediately posterior to the cement gland in *Xenopus* and from a crescent-like structure until stage 13 (*Xenopus*). This common cardiac progenitor cell population splits into two different lineages, also referred to as the first heart field (FHF) cell lineage and the second heart field (SHF) cell lineage [37,38]. In mouse, cells of the FHF contribute to the left ventricular myocardial cells, [39] and the two atria [40,41]. The SHF mainly forms the myocardium of the outflow tract (OFT) [42] but also contributes to the right ventricle [43]. In contrast, the *Xenopus* SHF only contributes to the OFT and the FHF forms into the single ventricle and the two atria of the heart. These two lineages can be distinguished by marker gene expression by E7.5 in the mouse and stage 24 in *Xenopus*. A widely used marker gene for the SHF also in *Xenopus* is the transcription factor Islet 1 [44,45], whereas Tbx 5 is one common marker of the FHF at this stage [46].

Eventually, the heart primodia merge and form the primary heart tube (*Xenopus* stage 31–33, mouse E8.0). Subsequently, the heart tube undergoes rightward looping (*Xenopus* stage 33–36, mouse E8.5) and the myocardium expands. At this point, the heart tube contains differentiated cardiomyocytes and begins to contract (*Xenopus* stage 35, mouse E8.5). After looping, the heart compartmentalizes into well-defined chambers (*Xenopus* stage 40–46, mouse E10.5). During this remodeling phase, the ventricular myocardium also undergoes trabeculation [15,32,33,37,47,48,49,50]. Considering these morphological as well as molecular similarities to the mammalian heart, *Xenopus* provides a valuable model for studying the underlying molecular mechanisms for the formation of the cardiovascular system and the progression of associated diseases.

## 4. *Xenopus* Models for Human Congenital Heart Defects

Aberrations in heart development are associated with numerous human congenital heart defects (CHDs) [55]. CHDs are the most common disorder in newborns with a prevalence of approximately 1% in live births and cause about 10% of stillbirths and spontaneous abortions [56,57]. Mutations in e.g., *NKX2.5, GATA* or *T-BOX* genes in patients are associated with CHDs such as atrial septal defects, DiGeorge Syndrome or Tetralogy of Fallot, to name a few. Several distinct characteristics of *Xenopus* emphasize its suitability for modeling CHDs when compared to other model systems. For example, gene function can be quickly analyzed due to the rapid embryonic development of *Xenopus*. As embryos develop in the absence of a functional circulation system, the onset of cardiovascular defects can be investigated *in vivo*. The three-chambered heart is not a disadvantage since the ventricle resembles the mammalian left ventricle [8] and allows for modeling of, e.g., hypoplastic left heart syndrome defects (HLHS). In contrast to zebrafish, the septated atria in the *Xenopus* heart allow for the study of atrial septal defects.

In recent years, more and more *Xenopus* models have emerged and the data from these models has complemented studies that used genetically modified mice as well as murine and human iPS cells (Table 2) [reviewed in 14]. In the following section, we briefly describe selected *Xenopus* models. For a more detailed description of the *Xenopus* models for human CHDs, we refer the reader to an excellent review from the Conlon group [14].

Atrial septal defects (ASDs) are one of the most common CHDs. An atrial septal defect is characterized by an incomplete separation of left and right atrium by atrial septum, which thereby allows oxygen-rich and oxygen-poor blood to mix. Several ASD patients have been identified with changes in the NKX2.5 protein [58,59] and its interacting partner, the zinc finger transcription factor GATA4 [60,61,62,63]. In patients, several point mutations in NKX2.5 have been identified. Overexpression of Nkx2.5 constructs carrying these mutations in *Xenopus* resulted in ASDs and conduction system defects that recapitulated the cardiac defects observed in patients [59,64]. Furthermore, knockdown of *gata4* via MO approach causes a strong reduction in heart precursor cells during cardiac specification and later defects in heart morphology. Furthermore, it has been proposed that GATA4, GATA5 and GATA6 function redundantly to regulate myocardial differentiation [65,66]. In mice, it has been shown that, for normal cardiac morphogenesis, the interaction between Gata4 and Tbx5 is critical [67]. Mutations in the T-box transcription factor *TBX5* account for more than 70% of patients with Holt-Oram Syndrome (HOS), which is characterized by ASDs, ventricular septal defects (VSDs) and cardiac conduction defects [68,69,70,71]. Overexpression of a dominant-negative Tbx5 protein in *Xenopus* inhibited heart tube formation. In contrast, loss of Tbx5 function caused unlooped heart tubes and decreased cardiac cell numbers due to decreased cell cycle progression. Thus, defects in cell proliferation probably cause cardiac defects, phenocopying defects of HOS patients [72,73,74].

Mutations in other members of the T-box family are accountable for other CHDs as well. Several mutations identified in *TBX20* contribute to familial CHD or congenital ASD [75,76,77,78,79]. In some Tetralogy of Fallot patients, *Tbx20* is upregulated [80]. Recent studies in *Xenopus* showed that Tbx20 does not affect cardiac specification and differentiation. However, depletion of Tbx20 resulted in unlooped heart tubes, pericardial edema, defects in chamber formation and reduced cardiomyocyte numbers [72]. Moreover, overexpression analyses in *Xenopus* embryos and animal caps revealed that TBX20 activity depends on its C-terminal domain [81]. Brown *et al.* demonstrated that TBX20 physically interacts with TBX5. Therefore, it is not surprising that the combined depletion of Tbx20 and Tbx5 in *Xenopus* resulted in more severe cardiac defects than loss of either one alone [72].

DiGeorge Syndrome or 22q11 deletion syndrome (del22q11DS) is characterized by symptoms such as Tetralogy of Fallot and cardio-facial abnormalities. The deletion includes the locus of the T-box gene *TBX1* [82,83,84]. Furthermore, several mutations in *TBX1* have been identified in patients with DiGeorge Syndrome phenotypes [85]. Overexpression of a dominant interfering mutant of *tbx1* in *Xenopus* resulted in unlooped hearts and pericardial edema [86,87] similar to the cardiac defects observed in patients.

Jacobsen syndrome (11q-) describes a rare condition caused by deletion of the distal part of chromosome 11 and includes many common CHDs, ventricular septum defect (VSD) as well as HLHS [88,89,90,91]. One gene in the deleted region is a member of the ETS family of transcription factors, ETS-1 [92]. A study in *Xenopus* discovered that Ets1 is required for cardiac neural crest and mesoderm formation. Depletion of Ets1 in cardiac neural crest tissue resulted in smaller, malformed OFTs analogous to defects of DiGeorge syndrome patients. Disruption of Ets1 via MO knockdown in the cardiac mesoderm delayed heart tube formation and impaired heart morphogenesis resulting in the loss of the three-chambered heart shape. Ets1-MO treated embryos had a single chamber heart with misshaped OFTs, an underdeveloped ventricle and a defective aortic arch formation resembling defects of HLHS patients. Due to reduced expression of *tbx1* and *mef2* in Ets1 morphants, it has been suggested that Tbx1 might be a direct target of Ets1 in the cardiac mesoderm and thus maybe partially responsible for the patients phenotype [93].

In 2013, novel gene variants in HLHS patients with left ventricular outflow tract (LVOT) conduction defects were identified in *MCTP2* (multiple C2-domains with two transmembrane regions 2) [94]. Gain and loss of Mctp2 function in *Xenopus* resulted in perturbed cardiac development with OFT defects in a dosage-sensitive manner. Mctp2 morphants exhibited pericardial edema, looping and OFT defects and failed to form endocardial cushion reminiscent for LVOT defects [94].

Another condition associated with various cardiac malformations is CHARGE syndrome [95], which, in most cases, is caused by heterozygous mutations or deletions of the *chromodomain helicase DNA-binding protein 7* (*CHD7*) [96,97,98,99,100,101]. CHARGE syndrome patients exhibit Tetralogy of Fallot, aortic arch and atrioventricular canal malformation [95]. Impairment of Chd7 in *Xenopus* led to cardiac defects reminiscent of CHARGE phenotype including OFT defects and truncus arteriosus abnormalities [102]. It has been demonstrated that Chd7 is crucial for the formation of migratory neural crest cells and regulates gene expression involved in neural crest cell and axon guidance [102,103]. Moreover, Chd7-deficient *Xenopus* embryos have reduced *semaphorin-3a* (*sema3a*) expression suggesting that disturbed *sema3a* signaling contributes to the pathogenesis of the CHARGE-related disorder Kallmann syndrome and possibly CHARGE syndrome itself [103].

## 5. Modeling GWAS Candidates Associated with CHDs in *Xenopus*

The first genome-wide association studies (GWAS) for coronary artery disease were published in 2007 [104,105,106]. Since then, GWAS pinpointed hundreds of genetic factors associated with cardiovascular diseases (CVDs) [107,108,109,110]. Also, whole-exome sequencing of patients and their relatives have identified a large set of mutations that are potentially disease causing. The large number of candidate genes derived by these methods generates the problem of separating mutations that are relevant from irrelevant mutations in the associated pathologies. Large-scale screens based on MO-mediated knockdown of gene function can be quickly performed in *Xenopus* with the aim to verify causal candidate genes [111,112].

Two recent examples shall highlight the suitability of *Xenopus* for GWAS studies. In 2011, a copy number deletion of the *N-acetylgalactosaminyltransferase 11* (*GALNT11*) gene was identified in a heterotaxy patient [108]. Depletion of Galnt11 in *Xenopus* resulted in abnormal cardiac looping. Knockdown as well as overexpression of human GALNT11 led to left-right patterning defects, mimicking primary ciliary dyskinesia in humans. The density of multiciliated cells was affected in these embryos caused by a deregulated Notch pathway [113].

Another GWAS associated the human zinc finger transcription factor *CASZ1* with hypertension and high systolic blood pressure [114,115]. In *Xenopus,* Casz1 is required for proper cardiovascular development [116,117] and, therefore, also for the heart’s proper physiological function. Depletion of Casz1 caused a subset of cardiac progenitor cells along the ventral midline to arrest. They are maintained as cardiac progenitors and fail to terminally differentiate into cardiomyocytes prior to heart tube formation [117]. Immunofluorescent analyses revealed Casz1 downregulation in cells reentering cell cycle and loss of Casz1 led to an increased mitotic index within cardiomyocytes. Thus, Casz1 regulates cell proliferation [118]. The authors hypothesized that Casz1 regulates cardiomyocyte growth and perhaps also their function, thereby influencing the physiological heart function. Casz1 directly interacts with the congenital heart disease 5 protein (CHD5), which is required for cardiac morphogenesis during heart looping and chamber formation. Depletion of CHD5 in *Xenopus* resulted in improper cardiomyocyte adhesion and deposition of basement membrane within myocardial tissue [119], which could potentially affect the blood pumping efficiency of the heart.

In summary, because of the anatomical, physiological and genetic similarities between *Xenopus* and mammals, this amphibian is ideally suited to investigate the molecular basis for vertebrate heart development and the progression of cardiac defects. MO-based large scale screening approaches as well as emerging techniques such as genome editing facilitate more in-depth analysis allowing *Xenopus* to thrive as an early model to investigate CHDs and GWAS candidate genes in the future.

## Figures and Tables

**Table 1:** Comparative timeline of events in cardiovascular development in different species. Based on [8,15,50,51,52,53,54].

Cardiovascular Event	Developmental Stage				
	*Danio Rerio*	*Xenopus Laevis*	*Mus Musculus*	*Gallus Gallus*	*Homo Sapiens*
cardiac progenitors	5 hpf	NF10	E6.5	HH4	CS7
heart field specification	12 hpf	NF12–NF14	E7.0–E7.5	HH5	CS8
migration to ventral midline	12–19 hpf	NF12–NF13	E7.5	HH7–HH8	CS9
primary heart tube formation	21–24 hpf	NF31–NF33	E8.0	HH9	CS10
onset of coordinated muscle contraction	22 hpf	NF35	E8.5	HH10	CS10
cardiac looping	30–36 hpf	NF33–NF36	E8.5–E10.5	HH10–HH24	CS13–CS17
onset of blood flow	30 hpf	NF35	E8.5	HH10	CS11
chamber formation	30 hpf	NF39–NF40	E9.5–E12.5	HH16/17	CS12–CS15
onset of ventricular trabeculaetion	48 hpf	NF41	E9.5	HH16	CS11
valvulogenesis	48 hpf	NF41–NF44	E9.5	HH21–HH36	CS15–CS18
atrial septation	none	NF44–NF45	E10.0–E14.5	HH16–HH36	CS14–CS18
ventricular septation	none	none	E9.0–E14.0	HH17–HH34	CS16–CS22

hpf: hour(s) post fertilization; NF: stages according to Nieuwkoop and Faber, 1956; E: embryonic day; HH: stages according to Hamburger and Hamilton, 1951; CS: Carnegie stages.

**Table 2:** Selected *Xenopus* models for human CHDs (see main text for details).

Disease	*Affected Genes*	*Xenopus* Model	Cardiovascular Phenotype
Atrial Septal Defects (ASD)	*gata4*	LOF	looping defects
	*nkx2-5*	GOF	cardiac conduction defects, ASD
Axonfeld-Reiger Syndrome	*pitx2*	GOF, LOF	looping defects and ASD
CHARGE Syndrome	*chd7*	GOF, LOF	neural cest migration and OFT defects
DiGeorge Syndrome	*tbx1*	GOF	looping defects
Holt-Oram Syndrome	*tbx5*	GOF, LOF	looping defects, reduced cardiomyocytes
Jacobsen Syndrome	*ets1*	LOF	OFT and aortic arch formation defects
LVOT obstructive defects, Hypoplastic left heart syndrome	*mctp2*	GOF, LOF	looping defects, OFT defects
Tetralogy of Fallot	*tbx20*	LOF	looping defects, reduced cardiomyocytes

ASD: Atrial Septal Defects, GOF: gain-of-function; LOF: loss-of-function; LVOT: left ventricular outflow tract; OFT: outflow tract.

## Abbreviations

The following abbreviations are used in this manuscript:

ASD: atrial septal defects
CHARGE: Coloboma, Heart defects, choanal Atresia, Retarded growth and development
CHD: congenital heart defects
CRISPR: clustered regulatory inter-spaced short palindromic repeats
DMZ: dorsal marginal zone
FHF: first heart field
HLHS: hypoplastic left heart syndrome
HOS: Holt-Oram syndrome
LVOT: left ventricular outflow tract
MO: morpholino oligonucleotides
OFT: outflow tract
SHF: second heart field
VMZ: ventral marginal zone
VSD: ventricular septal defects
